# “Disadvantaged patient populations”: A theory-informed education needs assessment in an urban teaching hospital

**Published:** 2019-11-28

**Authors:** Lindsay Baker, Emilia Kangasjarv, Beck McNeil, Patricia Houston, Stephanie Mooney, Stella Ng

**Affiliations:** 1 Department of Psychiatry, Faculty of Medicine, University of Toronto, Ontario, Canada; 2 Centre for Faculty Development, Faculty of Medicine, University of Toronto at St. Michael’s Hospital, Ontario, Canada; 3 The Wilson Centre, Faculty of Medicine, University of Toronto at Toronto General Hospital, Ontario Canada; 4 The Li Ka Shing Knowledge Institute, St. Michael's Hospital, Ontario, Canada; 5 Toronto Public Service and Leadership Development Programs, People, Equity and Human Rights Division, City of Toronto, Ontario, Canada; 6 Department of Anesthesia, Faculty of Medicine, University of Toronto, Ontario, Canada; 7 Department of Anaesthesia, St. Michael’s Hospital, Unity Health Toronto, Ontario, Canada; 8 Education Portfolio, St. Michael’s Hospital, Unity Health Toronto, Ontario, Canada; 9 Department of Speech-Language Pathology, Faculty of Medicine, University of Toronto, Ontario, Canada; 10 Centre for Ambulatory Care Education, Women’s College Hospital, Ontario, Canada

## Abstract

**Background:**

Recent calls in medical education and health care emphasize equitable care for disadvantaged patient populations (DPP), with education highlighted as a key mechanism toward this goal. As a first step in understanding potential education needs we wanted to better understand the DPP concept.

**Methods:**

Framed as a critical needs assessment, we used a critical discourse analysis approach to explore the meanings and effects of DPP. We analyzed transcripts from 15 focus groups with trainees, staff and patients.

**Results:**

We identified three main assumptions about DPP: 1) disadvantaged patients require care above what is normal; 2) the system is to blame for failures in serving disadvantaged patients; and 3) labeling patients is problematic and stigmatizing. Patients appreciated that the DPP concept opened up better access to care, but also felt ‘othered’ by the concept. As a result, patients felt they were not accessing the same level of care in terms of compassion and respect.

**Conclusion:**

We must define access beyond ability to receive services; access must also engender a sense of common humanity and respect. With this aim, we suggest three, theory-informed educational approaches to help improve care for DPP: 1) sharing authentic and varied stories; 2) fostering dialogue; 3) aligning assessment and educational approaches.

## Abstract (fr)

**Contexte:**

Des préoccupations récentes en éducation médicale et ensanté mettent l’accent sur les soins équitables dispensés auprès des Patients issus de Populations Défavorisés (PPD). Dans ce contexte, l’éducation est mise de l’avant comme un mécanisme clé dans l’atteinte de cet objectif. Comme première étape dans la compréhension des besoins potentiels en éducation, nous voulions mieux comprendre ce que recouvre le concept de PPD.

**Méthodes:**

Présentée comme une évaluation critique des besoins, nous avons utilisé une approche d’analyse du discours critique pour explorer les significations et les effets des PPD. Nous avons analysé les transcriptions de quinze groupes de discussion avec des stagiaires, du personnel et des patients.

**Résultats:**

Nous avons repéré trois suppositions principales au sujet des PPD : 1) les patients défavorisés nécessitent davantage de soins que la normale; 2) le système est à l’origine des défaillances à servir les patients défavorisés; et 3) l’étiquetage des patients est problématique et stigmatisant. Les patients ont aimé que le concept des PPD procure un meilleur accès aux soins, mais ils se sont sentis également « exclus » par les paramètres du concept. En conséquence, les patients estimaient qu’ils ne recevaient pas le même niveau de soins en matière de compassion et de respect.

**Conclusions:**

Nous devons définir l’accès au-delà de la capacité de recevoir des services; l’accès doit également engendrer un sens d’humanité commune et de respect. Dans ce but, nous suggérons trois démarches pédagogiques éclairées par la théorie pour aider à améliorer les soins aux PPD : 1) partager des histoires authentiques et variées; 2) promouvoir le dialogue; 3) aligner les démarches d’évaluation et d’enseignement.

## Introduction

Recent calls in medical education and health care have emphasized equitable care for patients experiencing disadvantage.^1^^,^^2^ Disadvantaged patient groups (individually and collectively) are increasingly considered in the development of hospital strategic plans and the social determinants of health (SDoH) are now common content in medical school curricula.^3^^–^^5^ SDoH are defined by the World Health Organization as the conditions in which people are born, grow, work, and live, and the broader set of systems that shape the conditions of daily life.^6^ At an individual level, SDoH such as housing, employment status, and working conditions impact people’s daily lives, determining their risk of illness and ability to access preventive and curative health care measures.^6^ At a societal level, inequities between groups of people shape how society is organized, often into hierarchies based on factors such as income, gender, and race.^7^ Where people sit in a social hierarchy ultimately affects their health and wellbeing in general.

In 2015, our hospital’s corporate strategic plan prioritized caring for disadvantaged patient populations – patients who are relegated to lower social status within the prevailing hierarchical structure of our societies. Our team was called upon to help develop a hospital-wide education approach to support the strategic priority of “transforming systems of care to ensure improvement in equitable access for all patients.” Underpinning our approach was a transformative paradigm of education. By paradigm of education, we are referring to different ways of conceptualizing the purpose and goals of education. Dominant cognitivist and behaviorist paradigms focus on changing behavior and teaching memorization and application of content knowledge, whereas a transformative paradigm focuses on shifting ways of seeing and inspiring social action. Therefore a transformative paradigm aligns with the ultimate goal of transforming systems.^8^

A necessary first step in designing any education initiative, is conducting a needs assessment. Given the identified need to attend to power when working toward equity in health,^9^ we used the critical conception of discourse as the theoretical frame for our needs assessment. By discourse we are referring to a language-based system of meaning, situated in an historical and cultural context. This system of meaning governs what we believe, and how we act. If we aim to transform systems, we first need to understand the discourses in our current system and what they are enabling or constraining.^10^^,^^11^ With a critical lens, discourses construct and give power to specific institutions, create roles for individuals to play in the system and make possible the existence of certain objects (material and conceptual). Without critical approaches to help examine discourses and how they influence what we believe and how we act, we risk merely perpetuating the status quo.^10^^,^^12^^,^^13^

Thus, we examined *disadvantaged patient populations* (DPP) as a dominant discourse in our organization with an eye to education needs and opportunities. We asked: How do people in our hospital community speak about DPP and what does this tell us about education needs and opportunities in relation to caring for DPP? By looking critically at the discourse of DPP, we can begin to understand the ways in which the dominant conception might limit actual change and identify meaningful ways forward through education.

## Methods

We conducted a critical needs assessment to explore the effects of DPP as a dominant discourse and what that tells us about education needs in our hospital. We do not presume that education will solve all the problems related to DPP, but we are interested in uncovering what educational needs may exist and be amenable to educational intervention. This study was approved by the St. Michaels’ Hospital (SMH) ethics committee.

## Setting

We situated our study within SMH , a hospital in the downtown core of Toronto Ontario, one of the world’s most ethnically diverse cities.^14^ Its geographic location and historical commitment to compassionate care for the disadvantaged led SMH to serve a diverse patient population. According to the 2015 Strategic Plan, “We care for people with severe and persistent mental illnesses and substance abuse issues, refugees, immigrants, vulnerable seniors, people with disabilities, and those challenged by other social determinants of health. We provide the homeless with a warm, safe place to recover after treatment in the Emergency Department.”

## Participants

A total of 70 participants agreed to participate in our needs assessment.

We recruited staff representing health disciplines, nursing, medicine, and other hospital staff through organizational gatekeepers (administrators of various departments) and trainees through the hospital’s student centre. All care providers (staff and trainees) learning and working at SMH were eligible to participate.

We recruited patients from the categories of disadvantage as named in the SMH strategic plan through partnerships with community organizations. These categories included: people experiencing mental health and addiction challenges, people who are homeless and underhoused, Indigenous peoples, new immigrants or refugees, and people across all sexual orientations, and gender identities. We also recruited patients falling outside these categories. Any patient living within the SMH catchment area and who self-identified with one of the categories was eligible to participate. Through our community partnerships, we identified key gatekeepers who could inform our recruitment and data collection and – through our partnership with them and engagement in a reflexive research approach^15^ – foster a safe and respectful engagement process. (see Table 1 for participant demographic details). Our reflexive approach is based upon published guidelines^15^ elaborated throughout our methods section and includes actions like inviting an Indigenous knowledge keeper to the focus group focusing on Indigenous health to help foster cultural safety.

## Data Collection

Three researchers conducted 15 one-hour semi- structured focus groups with care providers and patients. Care provider focus groups explored their understandings and practices relating to caring for disadvantaged patients, including probes about system influences. Examples of care provider focus group questions include: Who or what comes to mind when we say “disadvantaged patient”? How do you respond when caring for a disadvantaged patient? What enables you to care for these patients in the ways that you want to? We did not explicitly ask participants to list their perceived educational needs because we were focused less on content knowledge gaps and more on opportunities for humanistic and transformative education to support the goals of caring for DPP.^8^

Patient focus groups were held in community spaces familiar to participants. Patient focus groups sought and encouraged stories of general experiences with the healthcare system, including probes for positive and negative experiences, and what they wished healthcare providers knew. Patient focus group questions included: Are there any specific gaps you would like to see closed in terms of access to care? What would you like care providers to know about you?

Focus groups were digitally recorded and transcribed verbatim.

## Data Analysis

We analysed our focus group data using a thematic analysis,^16^ with the following questions: (1) What is the dominant discourse of DPP making sayable (i.e. socially acceptable, common, or ‘normal’) and unsayable? (2) What are the current ways to participate in the DPP discourse? (3) What activities are mobilized by the DPP discourse? These questions were informed by established theories about discourse, which tell us that language shapes and constrains social practices, knowledge and power. This way of questioning aligns with our transformative position that education is more than learning content knowledge; it is also about challenging assumptions and the status quo.^17^ In uncovering the ways people speak about DPP we presumed we would identify education needs.

We first identified and coded relevant meaning units and created analytic memos in response to the guiding questions. The coded meaning units were then synthesized into main themes, again in relation to the guiding questions. Bi-weekly meetings with the analysis team (LB, EK, SN) guided the reflexive analytic process.^15^ Analysis continued until the point of sufficiency, the point at which our coding was not leading to new insights.^18^

We used our findings as indicators of the remaining challenges to be addressed in relation to DPP in our organization. That is, we were looking for the assumptions embedded in the way DPP had operated and been acted upon as a starting point for continued improvement. Every innovation has unintended outcomes;^19^ it was these unintended, discursive outcomes that we framed as outstanding “needs” in our system, which transformative education approaches actively seek to address.

## Findings

We will present our findings in relation to our three main analytic questions, and from the standpoints of care providers and patients.

### What is sayable and unsayable in the current DPP discourse?

The DPP discourse was apparent in our dataset as three sets of assumptions: (1) disadvantaged patients require care above and beyond what is considered normal; (2) the system is to blame for failures in serving disadvantaged patients, and (3) labeling patients is problematic and stigmatizing.

***Disadvantaged patients require care above and*** ***beyond the norm*.** Care providers talked in terms of going above and beyond the call of duty in order to serve disadvantaged patients. This way of talking and thinking constructs a dichotomy between typical or regular patients and those experiencing disadvantage, and highlights exceptional effort and specialized expertise as requirements of care for these populations.

*I think, to some degree, we may put more effort, I would say, in people who are disadvantaged because, just as an example, the discharge will be more challenging*. (Care Provider, 04)

As a result, clinicians, in the DPP discourse, are said to be “good” care providers when they are willing and able to provide this additional care.

***The system is to blame for failures in serving*** ***disadvantaged patients*.** The DPP discourse makes sayable that no individual is at fault, but rather the problem lies within a system that struggles to meet the needs of all patients. Time constraints, lack of resources and support, and a convoluted, fragmented care system were highlighted as setting certain patients (and care providers) up for failure:

*I think there’s a tendency when people need more attention because of language barriers, cultural barriers, education barriers, or whatever it is to need a little extra time, but I think the system often responds by giving them less time*. (Care Provider, 08)

*I think it has to do with … the number of cases. They only give you a certain amount of time because there’s so many people to see and so many diagnoses to make and reports to fill out*. (Patient, 05)

***Labeling patients is problematic and stigmatizing***. The terminology surrounding ‘disadvantaged patients’ is resisted, to an extent, as problematic in and of itself. Care providers speak of the dangers of labeling, which they cautioned may further stigmatize, differentiate, and stereotype patients experiencing disadvantage:

*I know like we try to use terminology to kind of label a situation or a group of people so it’s easier to kind of capture information or the context, but sometimes by doing that, we kind of victimize the person and the individual or groups of people rather than look at the systemic issue*. (Care Provider, 03)

Patients felt essentialised (as if their personhood was lost and relegated to a category of disadvantage), and thus othered (positioned as different from and lesser than) by the DPP discourse. Although patients recognized that access to care was enabled by the DPP discourse, they also noted that this increased access was accompanied by negative associations. The DPP discourse's dehumanizing side effects created a call, by patients, to be seen as human beings, first and foremost, rather than being identified by their disadvantage:

*It seems like they forget that we [are] still human. They forget my name. Now I have a label of […]For some years I was even afraid to go to the doctor because … with those labels they just see an illness*. (Patient, 07)

### What are the current ways to participate in the DPP discourse?

Care providers participated in the DPP discourse as specialized DPP experts, advocates, and

system gatekeepers. Clinicians who address the ‘additional’ needs of disadvantaged patients are believed by colleagues to hold a particular set of values, cultural competencies, and expertise. They are positively framed as advocates and systems navigators for their patients, ensuring patients receive equitable and quality care:

*I spend a lot of time helping, trying to show them or help them to identify their own strengths, and to empower their own voice, trying to help them advocate for themselves and learn those skills so that might be a slightly different role that I get to take on versus other settings*. (Care provider, 02)

Care providers also act as gatekeepers, whether they are considered advocates or not. As gatekeepers they may either grant or deny access to resources from within or outside the healthcare system (e.g. forms for governmental benefits).

Patients participated in the DPP discourses either as desirable patients, or ‘invisible’ patients. Patients recognized that, at times, the system works against good care. Clinicians are busy and wait times are the norm for all. However, they explained that when you are seen as a “disadvantaged patient,” accessing humanistic care can become all the more challenging. They highlighted how disadvantaged patients are often seen as ‘difficult’ patients. And in order to be ‘a person worth caring for,’ patients had to perform or play the role of the ‘good patient’:

*I need to show that I’m not needy because if they get me on a bad day without makeup [...] in the Emergency room and whenever I go […] I have to look like I’m a presentable lady. Because if there is any sign that I could be on social assistance even or be the working poor, ooh…*(Patient, 05)

Participating in the DPP discourse required patients to be within one of the labeled groups listed in the strategic plan. That is, if you fit within one of the categories, you are able to access services. Therefore, patients who experience disadvantage beyond those six categories are in effect rendered invisible.

### What activities are mobilized by the DPP discourse?

We saw three continua of activities, each ranging from intended to unintended consequences, mobilized by the DPP discourse: (1) a comprehensive care approach that could become inadvertently myopic; (2) resource creation that could lead to competition for said resources; and (3) positive rhetoric coupled with both action and inaction.

***A comprehensive care approach that could become*** ***inadvertently myopic*.** The DPP discourse strives to provide more equitable, and thus comprehensive, care. However, when these well-intended goals become time and resource constrained, an unintended myopic approach to care can result instead. In this myopic approach, the disadvantage itself is targeted as if it is a singular impairment or diagnosis requiring treatment.

Targeting the disadvantage for treatment incidentally removes the complexity inherent in caring for a whole person. A consequence of myopic care – care that is well-meaning but too focused on disadvantage at the cost of caring for the whole person – is the inadvertent silencing of patients. Patients need a voice when their health and wellbeing is discussed; their knowledge and experience counts. Many stories demonstrated patients’ experiences of not being heard or believed, of false assumptions (and errors based upon these false, stereotypical assumptions), and de-humanizing interactions with care providers:

*He [the doctor] said, why are you here? And I said, I don’t feel good. And before he did anything, like temperature or anything, he said, well, you can’t get any narcotics. And I said, I don’t want any narcotics, that’s not why I’m here, I don’t take narcotics*. (Patient, 10)

***Resource creation that could lead to competition*** ***for said resources*.** When an organization focuses on disadvantage at a strategic level, attention and resources are often (re)directed toward this new priority. This added focus and funding offer beneficial opportunities and advancements for patients experiencing disadvantage; but these new resources have their limits, and competition for a limited pool of resources thus ensues. Advocates for particular disadvantaged patient populations are inadvertently positioned against one another for access to these limited resources. Demonstrating the greatest need and best investment thus becomes a part of the DPP discourse.

***Positive rhetoric coupled with both action and*** ***inaction*.** DPP as a discourse creates both internal and public messaging about the organization’s goals, which could be experienced as both helpful and as a tension. Language and messaging can shape perspectives; thus these forms of communication can help engender value for caring well for disadvantaged patients. However, tension also arises, between academic concepts associated with DPP (e.g. cultural competence) and the everyday practice of care providers.

Instead of oversimplistic and individualistic concepts like cultural competence, care providers pointed to systemic changes as top priority (as noted in the *What is sayable and unsayable in the current DPP discourse* section), described a recognition of the workarounds they engaged in everyday practice, and suggested a move toward shared responsibility as one way to improve care for disadvantaged patients. For example, they emphasized a need for collaborative relationships between hospital and community-based clinicians, which sometimes required taking an innovative or novel path:

*And you have to become more creative in finding resources or in finding ways to support them in the community. And at some point, as a team, I think at some point we have been very creative in looking at different ways, and sometimes taking the path less travelled*. (Care provider, 04)

Patients can see discrepancies between well- intended rhetoric espoused on posters and screens throughout the hospital, and the actualities of care they receive. They are aware that by supporting disadvantaged groups they may be unintentionally reproducing the disadvantage by singling them out. That is, they realize that the problems are complex and that efforts to help can inadvertently harm (e.g. by creating one-size-fits-all solutions for categories of patients, and perpetuating stigma):

*We wanted to be identified as separate. We wanted to have a voice for ourselves. Well they gave it to us. It didn’t kind of turn out the way we envisioned did it?* (Patient, 07)

## Discussion

The discourse of DPP – despite its espoused ideals of equity – serves to reinforce the social hierarchy that would need to be disrupted in order to achieve equity in health care. Without attention to power and social relations, categorizing patients into their most prominent sources of “disadvantage” risks positioning them as uniquely burdensome thus requiring additional effort from health professionals. This positioning separates the provider and patient rather than bringing them to a shared sense of understanding and responsibility. The categorization also further de-humanizes patients and leads providers to focus on discrete health or social issues rather than the whole complex person. While patients seem to recognize their disempowered position, providers may benefit from clearer awareness of their relationship to this disempowerment. With this awareness, they may be able to strive more toward sharing the responsibility rather than deferring blame to the system.^20^

Through a transformative paradigm of education,^8^ identifying dominant discourses related to DPP offers clear paths for educational recommendations. The purpose of transformative education is to shift orientations and perspectives.^8^^,^^21^^–^^23^ Therefore, identifying the dominant perspectives shows us where education can be helpful. Our discussion thus centers on the main problems identified in our needs assessment and opportunities that extant theory on transformative education and critical pedagogy offer in relation to these problems/needs. First, the DPP dominant discourse risks positioning disadvantaged patients as so distinct that they require exceptional effort. The unintended consequence of this positioning is a dehumanizing and ‘othering’ effect. Second, the DPP dominant discourse risks de-valuing the experiential and personal knowledge of both patients and providers, as corporate and strategic efforts can often unintentionally push aside the everyday knowledge and workarounds that are so core to truly compassionate and equitable care. And finally, the DPP dominant discourse risks narrowly defining equitable care and access to care such that the complexity and nuance they require is oversimplified. Thus assessment and evaluation outcomes for education risk falling into the trap of oversimplification and quantification that can reproduce inequity and poor access. Notably, access and equity must mean more than seeing a health provider and receiving medical treatment; they also mean being treated as valued human beings, just like any other patient.

The DPP “categories” at our organization align with current, popular education approaches that provide clinicians with the skills to identify the effect of social determinants on disadvantaged patients in a particular clinical encounter.^4^^,^^24^ These approaches, however, but do not equip clinicians with skills and virtues to understand and change the broader structural contexts in which the encounter takes place. Our empirical findings support the theoretical assertions made in extant litearture^4^^,^^20^^,^^24^ that teaching about the social determinants that cause certain individuals or groups to experience disadvantage, does not necessarily result in more equitable care. Our study saw care providers repeatedly citing ‘systems’ problems (i.e. knowledge of SDOH) for failures in serving disadvantaged patients, and experiencing little agency to enact change. Further, we saw DPP patients feeling singled out and dehumanized through such categorization and treatment. Sharma^4^ has suggested that teaching care providers to be aware of the SDOH, without teaching about the unequal distributions of wealth, power and privilege that contribute to health disparities, risks perpetuating this status quo.^4^ Sharma believes that when we categorize complex problems into DPP “categories” or lists of social determinants that affect people’s health, we risk practicing under the assumption that they are “natural” and not a result of societal structures over which we have some control that create these inequities.^4^ A critical approach to education is thus warranted.

### Future directions and limitations

Future research may need to examine the potential of critical approaches to education to address some of the needs and problems we saw in this needs assessment. Critical theory-informed educators argue that if we want care providers to see social determinants as actionable items that they can do something about, then we need to re-orient our education towards critical pedagogical approaches.^4^^,^^21^^–^^23^^,^^26^^–^^29^ The “critical” in “critical pedagogy” refers to a focus on questioning assumptions, attending to power relations, revealing the problems and opportunities these assumptions and relations may otherwise mask, and striving for transformation through positive change. The “pedagogy” in “critical pedagogy” refers to theories and practices of teaching and education.^30^ Based on findings from our needs assessment, we believe the following three education approaches may be suitable underpinnings for further study as opportunities to use education to improve care for patients experiencing disadvantage. These approaches are informed by work in critical pedagogy^21^^,^^23^ and are appropriate for academic hospitals in particular, wherein learning is largely experiential and workplace-based, and often pressed for time.

(1) Sharing authentic complex and varied stories in a range of safe, multi-media, and interactive formats. We suggest the theory-informed use of stories as a teaching approach.^31^^–^^35^ Using stories, in a complex and ethical manner, can address the sense of ‘othering’ – being made to feel distinct and less than – felt by patients who experience disadvantage. Stories have the potential to shift our narrow focus from disadvantage being a fixed characteristic, residing within a human being (as we saw in our needs assessment), to the view of a whole person within which ‘disadvantaged’ is but one label.

(2) Fostering dialogue instead of directives and discussion. We suggest a move toward dialogue more often than discussion. The educational difference between dialogue and discussion has been explained by Kumagai and Naidu.^37^ While discussion aims to arrive at a solution or consensus, dialogue aims to create questions and possibilities. It promotes the authentic exchange of ideas. “It begins in a safe learning space and invites learners to openly share their experiences without concern for judgment”.^23^ Rather than striving for a single, ‘best solution’ for a diverse group of unique patients, dialogue continually generates new questions and possibilities.^37^ Dialogue can potentially thus help us honour the experiential knowledge and complexity of patients and practitioners and, in combination with stories, can help address the problems of patients lacking voice and losing humanity in the health system, as identified in our findings.^37^

(3) Aligning assessment and evaluation with education approaches. An organization’s evaluation of staff and teams must align with its educational approaches;^38^ assessments and evaluations must honour the complexity of care. As described above, we need education that inspires a continual questioning of both professional and institutional practices to ensure no deliberate or inadvertent harm is being done. If stories and dialogue are the educational approaches, then the assessments of learning and evaluation of programs must align with these education approaches.

Many reasonable and practical factors in an organization drive staff evaluation towards a standardized – resources, transparency, actual and perceived fairness and equitability -- an approach which of course has its merits.^40^ Therefore, assessment and evaluative approaches that account for the complexity of care must find a balance between these potentially competing forces in the specific context of staff and trainees as learners and employees. This, we argue, is an area ripe for further study.

The local nature of our study, small sample sizes, and the fact that this inquiry was designed first and foremost as an organizational needs assessment limits its transferability to the broader literature yet allowed us to develop educational recommendations tailored to our specific context and potentially informative for others in similar circumstances. Future work should explore the relevance of our findings in other settings.

### Conclusion

Our needs assessment allowed us to explore the discourse of DPP as it is understood in our hospital context and its resultant educational needs, and our theory-informed approach to the needs assessment enabled us to identify educational approaches potentially well-suited to these types of educational needs. Based on the principles and practices of critical pedagogy, we identified and shared meaningful ways forward for education research to address the identified gaps. Our next steps involve exploring the implementation of our recommended education approaches within our organization. Critically, we need to find representative and paradigmatically aligned ways to meaningfully assess and evaluate this type of education.^8^

## Appendix A

**Table 1:** Participant details

Type of participant	Category	Number of participants
Care providers	Leaders	6
	Health disciplines	7
	Nursing	7
	Medicine	3
	Other	6
Patient	Mental health and addiction	5
	Homeless and Underhoused	9
	Indigenous	5
	Immigrant or refugee status	7
	Sexual orientation	1
	Gender identity	1
	General	4
Trainees	Health disciplines	3
	Nursing	2
	Medicine	4
Total		70

## References

[1] Sklar DP Disparities, Health Inequities, and Vulnerable Populations: Will Academic Medicine Meet the Challenge? Acad Med. 2018;93(1):1-3. doi:10.1097/ACM.0000000000002010. 29278585

[2] Sklar DP New Conversations: Justice, Disparities, and Meeting the Needs of Our Most Vulnerable Populations. Acad Med. 2017;92(11):1506-1507. doi:10.1097/ACM.0000000000001947. 29064993

[3] Dogra N, Reitmanova S, Carter-Pokras O Twelve tips for teaching diversity and embedding it in the medical curriculum. Med Teach. 2009;31(11):990-993. doi:10.3109/01421590902960326. 19909038 PMC2967223

[4] Sharma M, Pinto AD, Kumagai AK Teaching the Social Determinants of Health. Acad Med. 2018;93(1):25-30. doi:10.1097/ACM.0000000000001689. 28445214

[5] Chokshi DA Teaching about health disparities using a social determinants framework. J Gen Intern Med. 2010;25 Suppl 2Suppl 2:S182-5. doi:10.1007/s11606-009-1230-3. 20352516 PMC2847099

[6] World Health Organization About social determinants of health. https://www.who.int/social_determinants/sdh_definition/en/. Published 2018[Accessed October 3, 2019]

[7] Jones CP, Jones CY, Perry GS, Barclay G, Jones CA Addressing the Social Determinants of Children’s Health: A Cliff Analogy. J Health Care Poor Underserved. 2009;20(4A):1-12. doi:10.1353/hpu.0.0228. 20168027

[8] Baker L, Wright S, Mylopoulos M, Kulasegaram K, Ng S Aligning and applying the paradigms and practices of education. Acad Med. (In press). 10.1097/ACM.0000000000002693 30844933

[9] World Health Organization A Conceptual Framework for Action on the Social Determinants of Health.; 2010 https://www.who.int/social_determinants/corner/SDHDP2.pdf. [Accessed March 11, 2019].

[10] Foucault M, Sheridan A The Archaeology of Knowledge. 1st Americ. New York: Pantheon Books; 1972.

[11] Hodges BD, Martimianakis MA, McNaughton N, Whitehead C Medical education… meet Michel Foucault. Med Educ. 2014;48(6):563-571. doi:10.1111/medu.12411. 24807433

[12] Hodges BD, Kuper A, Reeves S Discourse analysis. BMJ. 2008;337:a879. 18687729 10.1136/bmj.a879

[13] Kuper A, Whitehead C, Hodges BD Looking back to move forward: Using history, discourse and text in medical education research: AMEE Guide No. 73. Med Teach. 2013;35(1):e849-e860. doi:10.3109/0142159X.2012.748887. 23259609

[14] UNESCO Toronto (Canada) A City’s good practice towards the elimination of discrimination. UNESCO Learning to Live Together. http://www.unesco.org/new/en/social-and-human-sciences/themes/fight-against-discrimination/coalition-of-cities/good-practices/toronto/. Published 2017 [Accessed October 3, 2019]

[15] Baker L, Phelan S, Snelgrove R, Varpio L, Maggi J, Ng S Recognizing and Responding to Ethically Important Moments in Qualitative Research. J Grad Med Educ. 2016;8(4):607-608. doi:10.4300/JGME-D-16-00384.1. 27777677 PMC5058599

[16] Nowell LS, Norris JM, White DE, Moules NJ Thematic Analysis. Int J Qual Methods. 2017;16(1). doi:10.1177/1609406917733847.

[17] Wodak R, Meyers M Methods for Critical Discourse Analysis. Sage; 2009.

[18] Ng S, Baker L, Cristancho S, Kennedy TJ, Lingard L Qualitative Research in Medical Education: Methodologies and Methods. In: Swanwick T, Forrest K, O’Brien B, eds. Understanding Medical Education: Evidence, Theory and Practice. 3rd ed The Association for the Study of Medical Education; 2019.

[19] Merton RK The Unanticipated Consequences of Purposive Social Action. Am Sociol Rev. 1936;1(6):894. doi:10.2307/2084615.

[20] Ventres W, Dharmamsi S, Ferrer R From Social determinats to social interdependency: Theory, reflection and egagement. Soc Med. 2006;11(2):84-89.

[21] Kumagai A, Lypson ML Beyond cultural competence: Critical consciousness, social justice, and multicultural education. Acad Med. 2009;84(6):782-787. 19474560 10.1097/ACM.0b013e3181a42398

[22] Freire P Pedagogy of the Oppressed. 20th Anniv. New York, NY: Continuum; 1993.

[23] Halman M, Baker L, Ng S Using critical consciousness to inform health professions education: A literature review. Perspect Med Educ. 2017;6(1):12-20. doi:10.1007/s40037-016-0324-y. 28050879 PMC5285284

[24] Kuper A When I say… equity. Med Educ. 2016;50(3):283-284. doi:10.1111/medu.12954. 26896013

[25] Dobson S, Voyer S, Hubinette M, Regehr G From the Clinic to the Community. Acad Med. 2015;90(2):214-220. doi:10.1097/ACM.0000000000000588. 25470309

[26] Kumagai AK From Competencies to Human Interests. Acad Med. 2014;89(7):978-983. doi:10.1097/ACM.0000000000000234. 24662200

[27] Boler M, Zembylas M Discomforting Truths: The Emotional Terrain of Understanding Difference. 12 2003:115-138. doi:10.4324/9780203465547-9.

[28] Landy R, Cameron C, Au A, et al Educational Strategies to Enhance Reflexivity Among Clinicians and Health Professional Students: A Scoping Study. Forum Qual Sozialforsch / Forum Qual Soc Res. 2016;17(3). doi:10.17169/FQS-17.3.2573.

[29] Kincheloe JL Critical Pedagogy Primer. P. Lang; 2008.

[30] Ng S, Baker L, Friesen F Teaching for Transformation. An Online Supplement. https://www.teachingfortransformation.com/whats-os/. Published 2018 [Accessed May 10, 2018].

[31] Charon R Narrative Medicine: A model for Empathy, Reflection, Profession and Trust. JAMA. 2001;286(15):1897. doi:10.1001/jama.286.15.1897. 11597295

[32] Frank AW Letting Stories Breathe: A Socio- Narratology. University of Chicago Press; 2010.

[33] Dennhardt S, Apramian T, Lingard L, Torabi N, Arntfield S Rethinking research in the medical humanities: a scoping review and narrative synthesis of quantitative outcome studies. Med Educ. 2016;50(3):285-299. doi:10.1111/medu.12812. 26896014

[34] Kumagai AK Perspective: Acts of interpretation: A philosophical approach to using creative arts in medical education. Acad Med. 2012;87(8):1138-1144. doi:10.1097/ACM.0b013e31825d0fd7. 22722358

[35] Kumagai AK, Wear D “Making Strange”: A role for the Humanities in Medical Education. Acad Med. 2014;89(7):973-977. doi:10.1097/ACM.0000000000000269. 24751976

[36] Phelan SK, Kinsella EA Picture This . . . Safety, Dignity, and Voice—Ethical Research With Children. Qual Inq. 2013;19(2):81-90. doi:10.1177/1077800412462987.

[37] Kumagai AK, Naidu T Reflection, Dialogue, and the Possibilities of Space. Acad Med. 2015;90(3):283-288. doi:10.1097/ACM.0000000000000582. 25426737

[38] Walsh A An exploration of Biggs’ constructive alignment in the context of work based learning. Assess Eval High Educ. 2007;32(1):79-87. doi:10.1080/02602930600848309.

[39] Titchkosky T Disability: A Rose by Any Other Name?“People-First” Language in Canadian Society*. Can Rev Sociol Can Sociol. 2008;38(2):125-140. doi:10.1111/j.1755-618X.2001.tb00967.x.

[40] Berg M Problems and promises of the protocol. Soc Sci Med. 1997;44(8):1081-1088. 9131732 10.1016/s0277-9536(96)00235-3

